# Influenza as a cause of severe acute respiratory infection through sentinel surveillance at national and provincial hospitals in Vietnam

**DOI:** 10.1186/2047-2994-4-S1-P13

**Published:** 2015-06-16

**Authors:** TP Nguyen, YTT Nguyen, TB Nguyen, TH Nguyen, HH Vu, MTQ Le, DN Tran, JM Partridge, JC Kile, TT Do, HT Nguyen

**Affiliations:** 1 Epidemiology, National Institute of Hygiene and Epidemiology, Hanoi, Vietnam; 2 Virology, National Institute of Hygiene and Epidemiology, Hanoi, Vietnam; 3 Bill and Melinda Gates Foundation, Seatles, USA; 4 Program, U.S. Centers for Disease Control and Prevention, Hanoi, Vietnam; 5 National Institute of Hygiene and Epidemiology, Hanoi, Vietnam

## Introduction

Since 2011 a National Influenza Surveillance System in Vietnam has conducted SARI surveillance to collect information and inform control and prevention strategies.

## Objectives

To determine the burden of influenza related SARI by gender and age.

## Methods

Selected patients met the WHO's SARI definition (an acute respiratory infection with history of fever or measured temperature ≥38C°; cough onset within the last 10 days; and requires hospitalization), were enrolled at four hospitals. Demographic, clinical data and one respiratory swab specimen were collected. Specimens were tested by PCR for influenza virus.

## Results

From March 2011 to April 2013, a total of 2,876 SARI patients were enrolled. The median age was 16 years (IQR 1-34 years). Of all enrolled patients, 1392 (48.4%) were children <15 years old and 1482 (51.5%) were male. 297 (10.3%) of 2,876 SARI cases were positive for influenza virus by RT-PCR. Positive rates by age group in years were 0 to <5 (26.9%), 5 to < 15 (1.7%), 15 to <25 (19.5%), 25 to <35 (25.3%), 35 to <45 (7.7%), 45 to <55 (6.7%), 55 to <65 (3.4%) and ≥65 (8.8%). Of the 297 influenza positive samples, 145(48.8%) were B, 85 (28.6%) were A/H3N2, and 67 (22.6%) were A/H1N1pdm09. Among the influenza positive patients, 45 (15.1%) were hospitalized in the ICU. At least one underlying health condition was reported by 57 (19.2%) influenza positive patients included smoking, lung disease, diabetes, liver disease, heart disease, hypertension, neurologic disease and pregnancy.

## Conclusion

Influenza virus infection was identified as a cause of SARI in hospitalized patients in Vietnam. As a vaccine preventable disease, it remains a burden to the healthcare system as well as a burden to the patient and family. The 0 to <5 and 25 to <35 age groups were more affected with influenza, accounting for 52.2% of influenza positive cases. Additional studies may help to better define risk factors related to infection and disease severity, evaluate the use of influenza vaccine in reducing disease burden in Vietnam.

## Disclosure of interest

None declared.

